# Validation of the Maximizing Tendency Scale in a Spanish Nursing Population

**DOI:** 10.3390/nursrep16010009

**Published:** 2025-12-25

**Authors:** Ricardo Tejeiro, Alberto Paramio, Serafín Cruces-Montes, Judit Santos-Marroquín, Antonio Romero-Moreno

**Affiliations:** 1 Department of Psychology, Liverpool John Moores University, Tom Reilly Building, Byrom Street, Liverpool L3 3AF, UK; r.a.tejeirosalguero@ljmu.ac.uk; 2 Department of Psychology, University Institute of Research in Social Sustainable Development (INDESS), University of Cádiz, C/República Saharaui, 12, 11519 Puerto Real, Spain; alberto.paramio@uca.es (A.P.); serafin.cruces@uca.es (S.C.-M.); 3 Professional College of Nursing of Cádiz, C/República Saharaui, 12, 11519 Puerto Real, Spain; judit.santos@gm.uca.es

**Keywords:** decision-making, maximization, MTS-7, nursing, reliability, validation

## Abstract

**Background:** In recent years, interest has grown in the study of the role of the maximization trait in situations of high uncertainty and high stakes. However, although up to 13 different scales have been proposed for its measurement, none of them have been translated and validated in the Spanish language. This study addresses this gap by adapting and validating the Spanish version of the 7-item Maximization Tendency Scale, a concise instrument designed to assess the tendency to maximize, which may offer practical advantages in terms of brevity and ease of administration compared to longer scales. **Objectives:** We aimed to adapt and evaluate the psychometric properties of the Spanish version of the MTS-7, examining its internal consistency and factor structure when applied to a Spanish sample. **Methods:** A sample of 213 active nurses from the province of Cádiz (Spain) (83.5% female) completed the translated version of the MTS-7 and completed the retest two weeks later. **Results:** Both Exploratory and Confirmatory Factor Analyses confirmed the unidimensional nature of the scale. Cronbach’s alpha coefficient was 0.78; the 2-week test–retest reliability Pearson correlation coefficient was 0.89; ICC was 0.78. **Conclusions:** The Spanish version of the MTS-7 possesses satisfactory psychometric properties and proves to have adequate reliability and validity. This scale may serve as a useful tool for studying decision-making under uncertainty among Spanish-speaking nurses.

## 1. Introduction

The “maximization” construct has attracted increasing interest among decision-making researchers. The concept was originally introduced by Simon [1], who argued that the human mind is constrained by cognitive limitations, making it unrealistic to achieve optimal decisions in most situations due to the overwhelming number of alternatives. As a result, people typically engage in “satisficing”—that is, they assess available options only until encountering one that sufficiently meets their objectives. Expanding on these ideas, Schwartz [2,3] proposed that the inclination to maximize is a relatively stable personality trait, with maximizing and satisficing representing opposing poles on a continuum. According to Diab et al. [4], this construct can be understood as a unidimensional trait reflecting a “general tendency to pursue the identification of the optimal alternative.” Within this framework, individuals who seek the best possible choice in decision-making are labeled “maximizers”, while those who settle for options that are sufficient or acceptable are termed “satisficers”.

Over the last twenty years, research on maximization and its role in decision-making has largely focused on consumer contexts [5,6] and academic settings [7,8], but has also extended to areas such as law enforcement [9] and the military [10]. A substantial body of evidence indicates that individuals with high maximization tendencies favor choices that involve numerous alternatives, adopt more analytical and rational decision-making approaches, and engage in distinct patterns of post-decisional evaluation [11]. Notably, chronic maximizers are more prone to procrastination [8], which can lead to decision inertia—a state characterized by avoidance, excessive deliberation, or difficulty executing decisions [12]. Maximizers often experience heightened fear of making suboptimal choices, leading to decision paralysis and delays in action. This avoidance behavior is further fueled by perfectionism, where maximizers delay completing tasks to ensure they meet their exceptionally high standards. Research suggests that the anxiety associated with potential regret over suboptimal choices contributes to procrastination, particularly when individuals face difficult decisions with many competing options [7,13]. Furthermore, research has shown that maximizers tend to experience lower levels of life satisfaction, happiness, optimism, and self-esteem [3].

Scholars differ significantly in how they conceptualize maximization, leading to the identification of up to seven distinct components: the aspiration to achieve the best outcome, setting high personal standards, actively searching for alternatives, experiencing difficulty in making decisions, the tendency to satisfice, feelings of regret, efforts to minimize outcomes, and reluctance to lower one’s standards [14,15]. This in turn has resulted in the development of various instruments for measuring the construct from the first scale developed by Schwartz [2]. Table 1 summarizes the characteristics of these instruments.

In their exhaustive review of what Cheek and Schwartz [14] refer to as the ‘befuddling literature’ on maximization, the authors contended that elements such as decision difficulty or regret should not be viewed as core components of maximization, but rather as its potential antecedents or consequences. They suggested a two-component model of maximization, understanding the concept as the pursuit of “[the] goal of choosing the best option through the maximization strategy of alternative search”. Their analysis of the tools available at the time led them to conclude that “the best measure of the goal of choosing the best is Dalal et al.’s MTS-7”. This is due to its superior psychometric performance compared to earlier versions, including the 9-item MTS. The MTS-7 exhibits a clear unidimensional structure with improved item-total correlations and acceptable reliability, offering a brief, conceptually coherent measure compared with earlier instruments [22].

The initial version of the MTS developed by Diab et al. [4] consists of 9 items, rated on a 5-point Likert scale ranging from 1 (completely disagree) to 5 (completely agree). The scale features items like “Whatever it takes, I always try to choose the best” and “I feel uncomfortable making decisions before knowing all my options.” The result is an overall score resulting from adding the item scores. The reliability (alpha coefficient) reported by the authors of the MTS is 0.80. The MTS-7 includes all items in the original scale except items 7 (“I am uncomfortable making decisions before I know all of my options”) and 8 (“Whenever I’m faced with a choice, I try to imagine what all the other possibilities are, even ones that aren’t present at the moment”). The authors reported that the scale had a unidimensional structure and an alpha reliability coefficient of 0.82, along with improved item-total correlations. Beyond being more concise and easier to administer than previous instruments, the MTS-7 has shown superior psychometric qualities when compared to earlier tools like the original Maximization Scale [3] or the Maximization Inventory [18]. By excluding dimensions not central to the construct—such as decision difficulty and regret—it offers a more streamlined and conceptually coherent measure [22]. This characteristic may be advantageous in time-pressured applied settings.

Overall, the MTS-7 is a brief, psychometrically robust unidimensional measure of maximizing tendency that can be applied across diverse cultural and occupational contexts and shows meaningful associations with satisfaction and well-being [26].

A Spanish adaptation of the MTS-7 is particularly relevant for nurses working in Spain, as it enables a precise and culturally appropriate assessment of maximizing tendencies in clinical decision-making. A validated Spanish version can support studies on how maximizing relates to stress, decision inertia, and job satisfaction in nursing, and it provides a common tool for integrating maximization measures into ongoing research on high-stakes decision-making in Spanish healthcare settings.

We plan to incorporate measures of maximization into a broader research programme on high-stakes decision-making in Spanish healthcare settings. However, all instruments summarized in Table 1 were originally developed in English-speaking countries, and, to the best of our knowledge, no published study has reported a validation of the MTS-7 in a Spanish (Spain) sample. Spanish-language work is available from Latin America—for example, the Maximizing Tendency Scale [4] adapted in Chile and the use of a Spanish version of the MTS-7 with Chilean participants [27,28,29]—but these do not constitute validations for the Spanish nursing context.

Cross-cultural research further underscores the need for country-specific validation. In a large study of 4690 individuals from 23 countries, Statman [30] showed that maximization covaries with risk tolerance and regret, patterns that are embedded in broader cultural dimensions such as uncertainty avoidance and individualism [31]. These findings suggest that measurement properties and correlates of maximization may not generalize automatically across cultures. Accordingly, the objective of the present study was to adapt and validate the Spanish version of the MTS-7 developed by Dalal et al. [22] in a sample of Spanish nurses, examining its internal consistency and factor structure. Based on previous work, we hypothesized a one-factor structure for the scale.

## 2. Materials and Methods

### 2.1. Design

This research was conducted as a cross-sectional descriptive observational study. We followed established recommendations for scale adaptation and validation in Health Sciences [32], including forward–backward translation, expert review with item-level and scale-level content validity indices (I-CVI, S-CVI/UA), pilot testing, and psychometric evaluation (EFA/CFA, reliability, and test–retest).

### 2.2. Participants

The Professional College of Nursing of Cádiz (Spain) was contacted by the authors and, after studying the research project, agreed to send the announcement for the study to all registered professionals in their regional area via email. Whilst the total number of registered nurses was 6081, the participation requirements included a minimum of 12 months of experience (no data as to how many met this requirement was made available to the authors). Because recruitment was conducted through the regional Professional College, participants were concentrated within the province of Cádiz (Andalusia) and should not be considered nationally representative. Nonetheless, the gender distribution (>80% female) mirrors the profession in Spain and supports local ecological representativeness. The announcement included a link to the study questionnaire (see below) as well as the authors’ contact information. Two hundred and thirty-seven active Spanish nurses agreed to participate, though 24 were excluded because they failed to complete the questionnaire or sign the consent form; the resulting sample was formed by 213 participants (Mean age = 24.54; SD = 3.31), most of them female (83.5%). This distribution mirrors the profession in Spain (e.g., >80% female; [33]) and thus supports ecological representativeness locally; however, it differs from Dalal et al.’s original MTS-7 validation sample (~63.8% female).

### 2.3. Instrument

The survey was developed and administered using online software from Qualtrics International Inc. Participants were recruited via a single email announcement circulated by the Professional College of Nursing of Cádiz to all registered nurses on its regional roster (N = 6081). The invitation included an open Qualtrics link and the authors’ contact details; no incentives were offered. Eligibility was screened within the survey as described below. It included questions to confirm the eligibility for the study and an item for the participant to indicate their gender. To be eligible, participants had to be licensed nurses currently working in Spain and have a minimum of one year of professional experience. This threshold was chosen because decision-making frameworks in the first year are still consolidating and are strongly shaped by supervision and onboarding protocols; excluding <1 year reduces construct-irrelevant variance in maximizing due to novice status rather than trait differences [34,35]. Nonetheless, we acknowledge that this may exclude highly driven early-career nurses; we therefore discuss potential bias toward higher ‘maximizing’ later in the Limitations.

We adapted and administered the 7-item scale by Dalal et al. [22], which is designed to measure the tendency to maximize versus satisfice in decision making. The 9-item scale by Diab et al. [4] is cited for context only; it was not administered. We selected the MTS-7 over the earlier 9-item MTS due to its clearer unidimensional structure, improved item-total correlations, and brevity—advantages for applied healthcare contexts. This instrument has been used in the USA, and at least in Canada, Austria, and Chile, on participants between the ages of 16 and 81 from various ethnic backgrounds. The seven statements are written in a 5-point Likert response format, where 1 means “strongly disagree” and 5 “strongly agree”. The Spanish version of Maximizing Tendency Scale [4] adapted to Chile by Moyano-Díaz and Mendoza-Llanos [28] showed an internal consistency of Cronbach’s alpha of 0.74 in working adults.

### 2.4. Procedure

**Figure 1:**
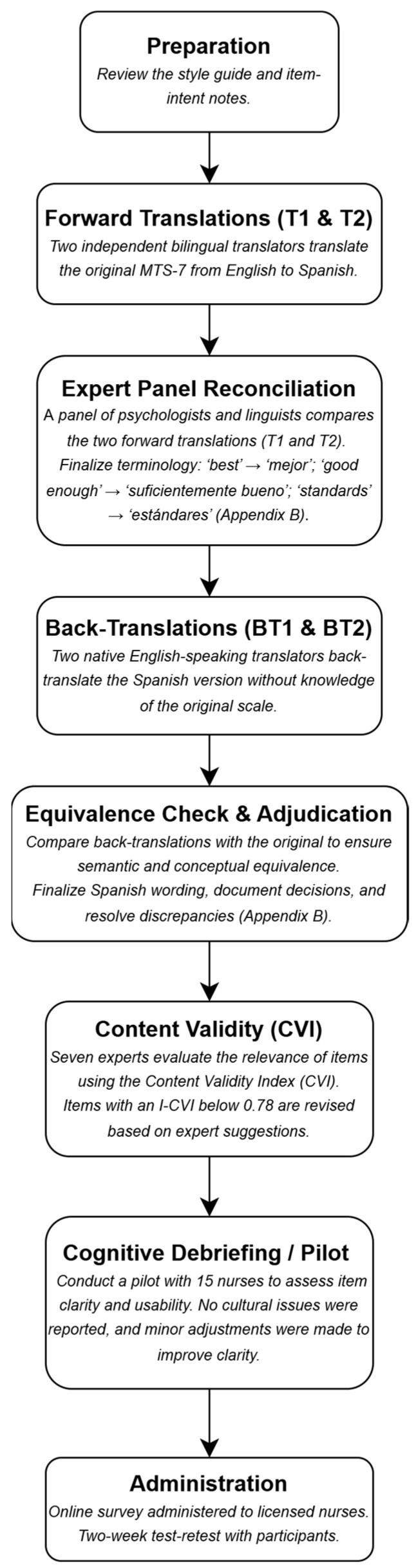
Flowchart of Translation and Validation Process (Spanish MTS-7).

The adaptation of the MTS-7 [22] adhered strictly to the World Health Organization’s recommendations for translating and adapting instruments [36] (Figure 1). Additionally, the process followed widely accepted international standards for the cross-cultural adaptation of self-report measures [37,38]. Initially, two bilingual translators independently carried out forward translations from English into Spanish. A synthesis of these translations was then reviewed by an expert panel composed of psychologists and linguists, who discussed discrepancies and reached consensus. Subsequently, two native English-speaking translators, who had no prior exposure to the original scale, independently conducted back-translations. This step aimed to verify both the semantic accuracy and the conceptual alignment of the translated version with the original. The expert panel compared the back-translations to the original English version, resolving inconsistencies and confirming equivalence. The item-reconciliation process can be followed in the Appendix B (Table A1). Discrepancies were logged and resolved by consensus, using item-intent notes and adjudication by a third senior translator when needed. For example, the term ‘best’ was debated (‘óptimo’ vs. ‘mejor’) and the panel retained ‘mejor’ to preserve colloquial clarity and comparability; ‘sufficiently good’ was standardized as ‘suficientemente bueno’ rather than ‘bastante bueno’; and ‘standards’ was rendered as ‘estándares’ (not ‘criterios’).

After completing the translation, a panel of seven experts specializing in psychology, psychometrics, and healthcare education carried out a content validation process. This assessment was conducted using the Content Validity Index (CVI) method, as proposed by Yusoff [39], and Polit and Beck [40]. Each expert rated the relevance/clarity of every item on a 4-point scale (1 = not relevant, 4 = highly relevant). Item-level CVI (I-CVI) was computed as the number of experts who scored the item 3 or 4 divided by the total number of experts (7). Scale-level CVI was calculated in two ways: (a) S-CVI/Ave, as the average of all I-CVIs, and (b) S-CVI/UA, as the proportion of items that obtained universal agreement (I-CVI = 1.00). Following Lynn’s decision rule [41], items with I-CVI < 0.78 were revised in wording and re-examined by the panel. Items with an I-CVI score below 0.78 were modified in accordance with the experts’ suggestions. Experts also provided qualitative comments, which were used to further refine item wording to enhance clarity, cultural relevance and professional appropriateness for the nursing context.

A pilot test was ultimately carried out with a group of 15 nurses to assess item comprehension and usability. Pilot participants were recruited from the same regional frame as the main sample and included nurses from acute hospital wards/ICU and primary care. Inclusion criteria matched the final study (≥1 year experience). Demographically and professionally, the pilot was intended to be broadly representative of the final sample to maximize content relevance during cognitive debriefing. Feedback from this phase led to minor rewording in two items to improve clarity without altering meaning. No item was reported as culturally inappropriate or difficult to interpret by the pilot participants. This finalized Spanish version of the MTS-7 (Appendix A) was then administered to the full study sample.

Participation required reading a participant information sheet online, ticking a box to consent to participate, and completing a questionnaire containing the translated version of the MTS-7. Participants were asked to self-generate a simple password for the retest, ensuring ease of recall and minimizing the chance of errors. The password was used to access the same survey link for both baseline and retest assessments. To ensure reliable matching of responses, participants were instructed to enter the exact same password in both assessments. In cases where discrepancies in the self-reported passwords occurred (e.g., typographical errors), those responses were excluded from the analysis. This approach ensured data integrity and that only valid retest responses were included in the study. Two weeks later, the Professional College of Nursing of Cádiz emailed an invitation to all its associates inviting those who completed the questionnaire to access a new link and answer again the items in the MTS-7. In addition to exclusions for missing data or lack of consent, we verified that each case met the eligibility criteria and checked for response pattern anomalies (e.g., straightlining). Response times were examined to detect abnormally fast completions (indicative of inattentive responding), though no cases were flagged for exclusion. No such anomalies were detected and only complete responses were analyzed (see Supplementary Materials).

### 2.5. Data Analysis

Statistical analyses were performed using SPSS version 29, licensed through the University of Cádiz. The distribution of quantitative variables was examined using the Kolmogorov–Smirnov–Lilliefors test [42] to assess normality. We did not conduct an a priori power analysis. Instead, we followed conventional rules of thumb for factor analysis (≥10 participants per item and ≥100 overall for short unidimensional scales) and continued recruitment until these minimums were exceeded. The final analyzed sample (n = 213) comfortably surpassed these thresholds. Missing data were handled via listwise deletion; only complete cases were analyzed. Internal consistency was evaluated through Cronbach’s alpha, corrected item-total correlations using Pearson’s correlation, and calculation of alpha values if individual items were deleted [43]. To assess the stability of participants’ scores, the intraclass correlation coefficient (ICC) with 95% CIs was applied [44]. Additionally, test–retest reliability over a two-week period was examined using Pearson’s correlation coefficient. We also computed I-CVI and S-CVI (Ave and UA) using the expert ratings described above [40,41]. I-CVI was obtained by dividing the number of experts who rated the item as 3–4 by 7; S-CVI/Ave was the mean of the seven item I-CVIs; S-CVI/UA was the proportion of items with I-CVI = 1.00.

Construct validity was examined through an exploratory factor analysis (EFA) and Confirmatory Factor Analysis (CFA):

Exploratory factor analysis: We examined construct validity using an EFA with Principal Axis Factoring and promax rotation, given the expectation of correlated indicators of a single latent construct. Sampling adequacy was evaluated with the Kaiser–Meyer–Olkin (KMO) index and Bartlett’s test of sphericity [45]. Salient loadings were defined as ≥0.40 for this short unidimensional scale. All EFA decisions (number of factors retained and rotation) were based solely on exploratory criteria.

Confirmatory factor analysis: To further evaluate model fit, we specified a one-factor model and assessed absolute (χ^2^/df, SRMR), parsimony (RMSEA with 90% CI), per Steiger and Lind [46]), and incremental fit indices (Tucker–Lewis Index [TLI] and Comparative Fit Index [CFI] by Bentler). Given its sensitivity to large sample sizes, the chi-square statistic was not used as a primary indicator of model fit [47]. A χ^2^/df ratio between 1 and 2 reflects good fit, while values between 2 and 3 suggest acceptable fit [48]. Good model fit is also indicated by SRMR values below 0.08, RMSEA values of 0.06 or less [49], TLI scores above 0.90 [50], and CFI values equal to or greater than 0.95 [51].

EFA and CFA were conducted on the same dataset due to the single-sample design; CFA results should therefore be considered preliminary and warrant replication in an independent sample.

### 2.6. Ethical Statement

The research received approval from the Health and Life Sciences Research Ethics Committee (Psychology, Health and Society) at the University of Liverpool (reference: 9834, approval date: 12 April 2021) and was carried out in accordance with the principles outlined in the Declaration of Helsinki [52]. It also complied with Spanish Organic Law 3/2018 of 5 December on the Protection of Personal Data and Guarantee of Digital Rights, as well as with Regulation (EU) 2016/679 of the European Parliament and Council, dated 27 April 2016.

All participants received an information sheet along with the questionnaire, and were assured that their responses would remain anonymous and confidential. Informed consent was obtained from each participant, and no form of compensation was provided for their involvement in the study.

## 3. Results

### 3.1. Descriptive Data

The mean overall score of the Spanish version of the MTS-7 in the sample was 26.1 (SD = 4.2). This result is consistent with those reported in previous research using the MTS-7 with university populations ([22], M = 25.6), suggesting that the Spanish version elicits comparable responses. Nevertheless, the mean was slightly higher than in Dalal et al.; differences in sample composition and context could contribute, but this requires direct testing. Table 2 shows the mean and standard deviation for each item.

### 3.2. Content Validity

Overall, the expert ratings showed high agreement. I-CVI values for the final Spanish version ranged from 0.86 to 1.00, indicating that all items met or exceeded the minimum acceptable level for panels of 6–10 experts. The scale-level indices were S-CVI/Ave = 0.93 and S-CVI/UA = 0.71, which, according to commonly used benchmarks, reflect excellent content validity for a short, unidimensional instrument. One item received slightly lower initial agreement because of wording preferences related to professional register in nursing, but the expert suggestions were incorporated and the revised wording achieved an I-CVI above the 0.78 threshold. Taken together, these quantitative indices and the qualitative comments from the panel support the semantic, conceptual, and contextual adequacy of the Spanish MTS-7.

### 3.3. Internal Consistency and Stability

The Cronbach’s alpha coefficient for the full 7-item scale was α = 0.78, which, according to Field [45], reflects an acceptable level of internal consistency given the number of items. Table 2 presents the corrected item-total correlation values and the alpha coefficient obtained if each item were removed. The analysis showed that all items demonstrated adequate correlations with the total score. Test–retest reliability over a two-week interval yielded a Pearson correlation coefficient of 0.89 (*p* = 0.000). The intraclass correlation coefficient (ICC), calculated using the average measures with between-measure variance excluded from the denominator, was 0.78 (95% CI = 0.72–0.83; *p* = 0.000).

### 3.4. Construct Validity

Exploratory factor analysis: Bartlett’s test of sphericity (χ^2^ = 316.334; df = 21; *p* = 0.000) indicated that the correlation matrix significantly differs from an identity matrix, and the result of the KMO test (0.79) was very close to the 0.80 threshold indicating that sampling is adequate for a factor analysis [53]. We carried out a Principal Axis Factor analysis using promax rotation. The components loadings and communalities are presented in Table 3. A single-factor solution accounted for 41.92% of the total variance, with all item loadings reaching or exceeding the 0.40 threshold.

**Figure 2:**
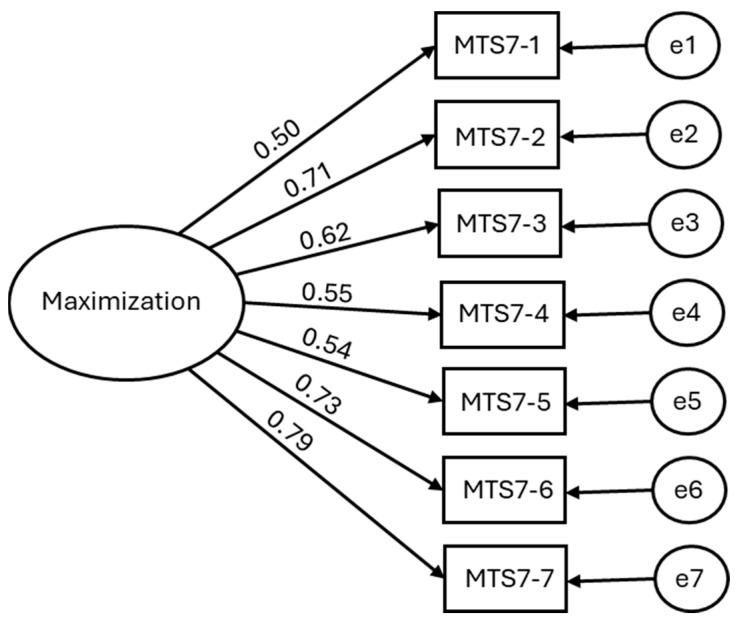
Confirmatory factor analysis model of the Maximization construct with standardized coefficients.

Confirmatory Factor Analysis: the normed chi-square value (χ^2^/df = 2.66) indicated that the one-factor model provided an acceptable fit to the data. The one-factor CFA measurement model (standardized solution) is depicted in Figure 2. All the other indices revealed a good model fit: SRMR = 0.05, TLI = 0.92, CFI = 0.94, RMSEA = 0.09 [90% CI: 0.06 to 0.13]. Given the very small degrees of freedom of one-factor short scales, RMSEA is known to be upward-biased and overly strict [47]. RMSEA values above 0.06 are typically considered unacceptable, but in models with low degrees of freedom (e.g., small sample sizes or models with few parameters), RMSEA can be biased upward, leading to higher values such as 0.09. This is a limitation of the model fitting process; therefore, we emphasize the use of alternative fit indices, such as SRMR, CFI, and TLI, which are more reliable in the context of low degrees of freedom [54].

Together with (i) EFA/CFA convergence, (ii) all loadings ≥ 0.40, and (iii) low residuals, the overall pattern supports acceptable fit of the one-factor model despite the RMSEA = 0.09. According to standard criteria [49,51], values of SRMR ≤ 0.08, TLI ≥ 0.90, and CFI ≥ 0.90 indicate good fit. Although the RMSEA exceeded the commonly preferred threshold (0.06), it tends to be upward-biased with few degrees of freedom. Accordingly, we interpret it with caution and place greater weight on SRMR, CFI, and TLI, which indicated an acceptable fit for a one-factor model.

## 4. Discussion

The primary objective of this study was to translate the MTS-7 [22] into Spanish and evaluate its psychometric properties within a Spanish-speaking sample. Consistent with the findings reported by Dalal et al. [22], the factor analyses supported the adequacy of a one-factor structure for the scale. This is also the result obtained by the authors of the original 9-item scale [4], and the factor can be considered as representing the goal of choosing, as opposed to the strategy of choosing [14]. This alignment with previous validation studies adds convergent evidence relative to the original instrument, while broader cross-language generalizability should be examined across independent samples.

The Spanish version of the MTS-7 displayed good psychometric properties. Cronbach’s alpha coefficient was slightly smaller than in the reference studies but still within the 0.70 to 0.90 acceptable range [55] and more than satisfactory for the small number of items [45]. Moreover, the strong test–retest reliability observed over a two-week period confirms the consistency of the scale scores over time, while the solid ICC values indicate that the scale demonstrates high overall test–retest stability (Pearson r and ICC).

Although these psychometric properties are encouraging, it is important to contextualize them within the unique challenges of decision-making in nursing practice. Research has consistently shown that nurses make frequent and high-stakes decisions under conditions of uncertainty, ambiguity, and time pressure [35,56]. In these contexts, maximizing tendencies may influence not only the quality of decisions but also the emotional burden and cognitive workload associated with decision-making [57]. Therefore, our findings suggest the potential value of exploring in future studies how maximization interacts with nursing decision-making processes in real-world settings.

Despite the abundance of research on maximization in general populations, its implications for healthcare professionals—especially nurses—remain underexplored. It is plausible that maximizing tendencies may influence clinical decision-making, particularly under time pressure, ambiguity, or emotional demands, although this relationship requires further empirical support. In such contexts, professionals with high maximizing tendencies might experience delays or elevated stress due to their drive to identify the optimal course of action. Indeed, recent studies suggest that maximizers may require more information and more time to feel confident in their decisions, a pattern observed in critical contexts like triage during mass casualty incidents [58]. This hesitation could translate into delays in clinical interventions, which are particularly consequential in acute care [35,56]. However, our study did not assess clinical performance, so this remains a hypothesis for future research.

Our study has certain limitations to consider. First, we did not perform criterion validity analyses. We did not correlate Spanish MTS-7 scores with other maximization-related scales or with conceptually related constructs, nor did we include behavioural indicators of decision performance such as search effort, choice quality, or decision time, which previous research has linked to maximization scores [2,4,22]. Future research should incorporate such external criteria and examine predictive relationships between maximization and clinical or decision-making outcomes, especially in high-stakes nursing contexts [56,57].

Our sample may also be a source of limitations. First, it was made up solely of experienced nurses. In contrast to many other professions, the constantly evolving and unpredictable context of healthcare demands that nurses possess strong decision-making skills to effectively address the needs of patients, their families, and fellow professionals within the system. In other words, “they must be able to sift and synthesize information, make decisions and adequately implement these decisions to solve problems in the context of a multidisciplinary team” [59]. Experienced nurses often rely on a blend of analytic and intuitive decision-making, developed through pattern recognition and situational awareness [34,35]. As such, their maximization tendencies might manifest differently compared to novice nurses, who tend to favor more analytical and linear approaches [57]. Therefore, generalizability to other Spanish-speaking healthcare workers (e.g., physicians, paramedics, nursing assistants) and to non-Spanish-speaking settings remains uncertain. Future multi-site studies with measurement invariance testing across professions, countries, and genders are warranted. It is possible that other populations’ experiences with difficult decision-making situations may respond differently to the questionnaire. Furthermore, 83.5% of participants were women. Whilst this is a faithful representation of the gender balance in the profession in Spain (in 2019, 84.2% of nurses were female [33]), it differs from the study by Dalal et al. [22], where females were also majority but to a lesser degree (63.8%). Finally, only 16% of participants took part in the retest, which in addition to the small resulting number carries on the possibility of some form of self-selection. This low completion rate may bias stability estimates if respondents were more conscientious or engaged, traits that often correlate with survey compliance, potentially inflating test–retest coefficients. Although anonymity prevented a formal comparison between re-testers and non-re-testers, we interpret test–retest results with caution and recommend that future studies implement strategies (e.g., reminders/incentives, scheduled re-contacts) to improve re-test uptake. Finally, RMSEA was slightly above conventional cut-offs (0.09); because this index tends to be inflated in models with few degrees of freedom [60], we interpreted it with caution and relied primarily on SRMR, CFI, and TLI, which indicated acceptable fit for the one-factor model.

Despite these limitations, our study contributes valuable evidence supporting the use of the MTS-7 in Spanish-speaking nursing populations. By validating the scale in this professional context, we provide a tool that can be used in future research to explore potential relationships between decision-making tendencies, stress management, and clinical performance. Given the complex and dynamic environments where nurses operate, future research should examine how maximization interacts with factors such as team support, institutional culture and the use of decision aids [35,56]. Such studies could inform interventions to optimize decision-making and reduce decision fatigue in nursing practice.

The MTS-7 could be used to assess decision-making tendencies in nurses and to inform hypothesis-driven training or simulation design; whether such uses reduce stress, decision inertia, or burnout should be tested in future studies.

## 5. Conclusions

The Spanish adaptation of the MTS-7 demonstrated adequate psychometric properties in a sample of nurses working in Spain, replicating the one-factor structure and acceptable internal consistency and test–retest reliability reported in the original validation [22]. This unidimensional focus addresses previous concerns about multidimensionality and construct overlap in earlier maximization measures and provides a brief tool suitable for use in Spanish-speaking nursing populations.

The availability of a validated Spanish version of the MTS-7 will facilitate cross-cultural research on maximizing and decision-making and allow future studies to examine how maximizing tendencies relate to decision satisfaction, stress, and clinical performance in healthcare and other professional settings. Our findings support the use of the MTS-7 as a research and educational instrument rather than a diagnostic or personnel selection tool; prospective studies should evaluate whether interventions informed by maximizing tendencies can improve decision timeliness, reduce decision-related stress, or enhance the quality of care.

## Figures and Tables

**Table 1:** Summary of existing maximization-related scales.

Scale	Authors	No. of Items	Maximizing Dimensions *	Satisficing Dimensions *
Maximization Scale	Schwartz [2]; Schwartz et al. [3]	13	1	0
MS-6	Nenkov et al. [16]	6	3	0
Maximizing Tendency Scale	Diab et al. [4]	9	1	0
Modified Maximizing Scale	Lai [17]	5	1	0
Maximization Inventory	Turner et al. [18]	34	2	1
Revised MS	Weinhardt et al. [19]	7	3	0
Revised MTS	Weinhardt et al. [19]	6	3	0
Relational Maximization Scale **	Mikkelson & Pauley [20]	15	1	0
Refined Maximization Scale	Richardson et al. [21]	10	3	0
MTS-7	Dalal et al. [22]	7	1	0
Decision Making Tendency Inventory	Misuraca et al. [23]	29	2	2
Scale of Friendship Maximization **	Newman et al. [24]	16	3	0
Revised Relational Maximization Scale **	Mikkelson & Ray [15]	14	1	0

* According to Misuraca and Fasolo [25]. ** Domain-specific scales.

**Table 2:** Descriptive Statistics and Item Analysis (n = 213).

	Mean	SD	Scale Mean if Item Deleted	Scale Variance if Item Deleted	Corrected Item-Total Correlation	Cronbach’s Alpha if Item Deleted
MTS7-1	4.32	0.80	22.04	13.64	0.415	0.765
MTS7-2	4.15	0.77	22.21	12.91	0.586	0.736
MTS7-3	3.59	0.89	22.77	12.96	0.467	0.756
MTS7-4	3.95	0.86	22.41	13.08	0.463	0.757
MTS7-5	3.37	0.89	22.99	13.43	0.381	0.773
MTS7-6	3.39	1.05	22.97	11.44	0.590	0.730
MTS7-7	3.59	0.94	22.76	11.84	0.619	0.724

**Table 3:** Exploratory factor loadings and communalities for the Spanish MTS-7 (n = 213) *.

	Loading **	Communalities
MTS7-1	0.58	0.34
MTS7-2	0.74	0.55
MTS7-3	0.62	0.39
MTS7-4	0.61	0.37
MTS7-5	0.52	0.27
MTS7-6	0.74	0.55
MTS7-7	0.76	0.58

* Extraction method: Principal Axis Factoring with promax rotation; explained variance = 41.92%. ** Only loadings ≥ 0.40 were considered salient.

## Data Availability

The data that support the findings of this study are available on request from the corresponding author. The data are not publicly available due to the restrictions as specified in the Organic Law 3/2018, of 5 December, on Protection of Personal Data and Guarantee of Digital Rights Law (Spain).

## Supplementary Materials

The following supporting information can be consulted at http://doi.org/10.17605/OSF.IO/YBV74 (accessed on 21 December 2025), Dataset with all the variables of the study sample.

## Public Involvement Statement

No public involvement in any aspect of this research.

## Guidelines and Standards Statement

This study was conducted and reported in accordance with the STROBE (Strengthening the Reporting of Observational Studies in Epidemiology) guidelines for observational research.

## Appendix A

### MTS-7 Spanish

Por favor, indique su grado de acuerdo con cada una de las siguientes afirmaciones referidas a usted mismo/a en general utilizando una escala de 5 puntos (1 = Totalmente en desacuerdo a 5 = Totalmente de acuerdo):

1. Cueste lo que cueste, siempre intento elegir lo mejor.
2. No me gusta tener que conformarme con lo “suficientemente bueno”.
3. Soy un/a maximizador/a.
4. Haga lo que haga, mantengo siempre los estándares más altos para mí mismo/a.
5. Siempre aguardo a que se presente la mejor opción, tarde lo que tarde.
6. Nunca me conformo con la segunda mejor opción.
7. Nunca me conformo con menos.

## Appendix B

**Table A1:** Item-level reconciliation.

Item #	Original (EN)	Forward T1 (ES)	Forward T2 (ES)	Reconciled Spanish (Final)	Back-Trans 1 (EN)	Back-Trans 2 (EN)
1	No matter what it takes, I always try to choose the best.	Cueste lo que cueste, siempre intento elegir lo óptimo.	Cueste lo que cueste, siempre intento elegir lo mejor.	Cueste lo que cueste, siempre intento elegir lo mejor.	Whatever it takes, I always try to choose the best.	At all costs, I try to choose the best option.
2	I don’t like having to settle for ‘good enough’.	No me gusta tener que conformarme con lo ‘bastante bueno’.	No me gusta tener que conformarme con lo ‘suficientemente bueno’.	No me gusta tener que conformarme con lo ‘suficientemente bueno’.	I don’t like having to settle for something that is ‘good enough’.	I dislike settling for what is merely ‘good enough’.
3	I am a maximizer.	Soy un maximizador.	Soy un/a maximizador/a.	Soy un/a maximizador/a.	I am a maximizer.	I consider myself a maximizer.
4	Whatever I do, I always maintain the highest standards for myself.	Haga lo que haga, mantengo siempre los criterios más altos para mí mismo/a.	Haga lo que haga, mantengo siempre los estándares más altos para mí mismo/a.	Haga lo que haga, mantengo siempre los estándares más altos para mí mismo/a.	Whatever I do, I always keep the highest standards for myself.	In anything I do, I maintain the highest standards for myself.
5	I always hold out for the best option, no matter how long it takes.	Siempre espero a que aparezca la mejor opción, tarde lo que tarde.	Siempre aguardo a que se presente la mejor opción, tarde lo que tarde.	Siempre aguardo a que se presente la mejor opción, tarde lo que tarde.	I always wait for the best option to appear, no matter how long it takes.	I hold out for the best option, however long it takes.
6	I never settle for the second-best.	Nunca me conformo con la segunda mejor alternativa.	Nunca me conformo con la segunda mejor opción.	Nunca me conformo con la segunda mejor opción.	I never settle for the second-best option.	I do not accept the second-best.
7	I never settle for less.	Nunca me conformo con menos.	Nunca acepto menos de lo mejor.	Nunca me conformo con menos.	I never settle for less.	I never accept less.
